# Comparison of Different Analgesia Drug Regimens for Pain Control During Extracorporeal Shock Wave Lithotripsy for Renal Stones: A Randomized Control Study

**DOI:** 10.7759/cureus.1195

**Published:** 2017-04-26

**Authors:** Muhammad Waqas, Amna Butt, Mohammad Ayaz Khan, Ijaz Khan, Imad-ud-din Saqib, Tariq Hussain, Saeed Akhter

**Affiliations:** 1 Department of Urology, Shifa International Hospital, Islamabad, Pakistan; 2 Department of Urology, Pakistan Kidney Institute, Islamabad, Pakistan; 3 Department of Plastic Surgery, Shifa International Hospital, Islamabad, Pakistan; 4 Lithotripsy Department, Shifa International Hospital, Islamabad, Pakistan

**Keywords:** analgesia, extracorporeal shockwave lithotripsy, lidocaine gel, naproxen sodium, pain score

## Abstract

**Introduction:**

With the increased use of extracorporeal shock wave lithotripsy (ESWL), the management of urolithiasis has become much convenient for the patients and the health care professionals alike. However, associated with the procedure is the common complaint of pain. No agreed upon pain management strategy has yet been developed for the procedure. We compared the effect of different analgesia drug regiments for pain control.

**Methodology:**

A randomised controlled trial was carried out in Shifa International Hospital from between July 2015 to January 2016. A total of 135 patients were divided into three groups; group A received 30 g lidocaine 2% gel applied locally on corresponding lumber area 30 minutes before the procedure, group B received oral naproxen sodium 550 mg 45 minutes before the procedure, and group C received both oral naproxen and lidocaine gel. Patients were supplemented with intravenous nalbuphine during the procedure. The pain was assessed with 0-10 visual analogue scale. Both pre-procedure and post-procedure pain score was measured.

**Results:**

Among 135 patients, 105 (77.8%) were male and 29 (21.5%) were female with mean age of 38.7 ± 1.31 years. There was no difference of mean pain score or need for supplemental intravenous nalbuphine between groups B and C but there was significantly decreased mean pain score and need for supplemental intravenous nalbuphine in groups B and C in comparison with group A.

**Conclusion:**

The use of oral naproxen sodium with or without the addition of lidocaine gel during ESWL is a promising option for pain management during the procedure with significant improvement in comparison with lidocaine gel alone.

## Introduction

The introduction of extracorporeal shock wave lithotripsy (ESWL) has revolutionized the treatment of urolithiasis due to it being non-invasive in nature, cost effective, and its associated reduced hospitalization time along with lower morbidity [1-2]. The impact of shock waves causes pain in the majority of the patients, requiring intervention for analgesia or sedation. It is important to keep the patients pain free to get the maximum possible success rates.

Opioids are used most commonly for pain relief in ESWL but the associated side effects require patient monitoring and delayed hospital stay [3-4]. In some studies, nonsteroidal anti-inflammatory drugs (NSAIDs) have also shown promising results with regards to pain control without the side effects associated with opioids [1, 5]. Local analgesia gels have a controversial role in relieving pain in ESWL [1, 6].

There are as yet no guidelines for pain relief during ESWL [1, 4]. In this study, our intent was to compare the effect of local analgesia gel, oral NSAIDs, and their combination on pain during lithotripsy.

## Materials and methods

We conducted a randomized control trial at the Department of Urology, Shifa International Hospital Islamabad. After institutional review board approval and written informed consent from the patients, we included all the patients who underwent ESWL from July 2015 to January 2016.

Patients were included only if they were older than 18 years, had renal stones, and understood the pain scoring system. All those patients who had any of the following were excluded; 1) history of chronic use of analgesia, 2) allergy to any of the used medications, 3) those having ureteric stones, 4) serum creatinine > 1.4, 5) pregnant patients, 6) those having gastric ulcer disease, 7) not willing to participate, 8) having any coagulopathy, and 9) active urinary tract infection.

Patients were randomized into one of three groups by lottery method, groups A, B and C. Patients in group A received 20 g lidocaine 2% gel application on the lumbar region corresponding to the entry site of the shockwaves, 30 minutes prior to the procedure, patients in group B received oral naproxen sodium 550 mg 45 minutes prior to the procedure while patients in group C received both locally applied 2% lidocaine gel and oral naproxen sodium 550 mg per oral at the times mentioned previously.

We used Modulith SLX lithotripter 4th generation Storz (Kennesaw, GA, USA) medical equipment for ESWL. Before entering the procedure room, patients were explained the procedure along with the visual analogue pain scale. Patients were asked to rate their pain on a scale from 0 (no pain) to 10 (maximum pain). During the procedure, pulse, blood pressure, and oxygen saturation were monitored. All patients were assessed by the same physician who was blinded to the patient group assignment. Analgesia was augmented with intravenous nalbuphine on patient demand during the procedure. Patients were monitored for ventilatory depression, decrease in oxygen saturation, nausea, vomiting, drowsiness, and hypersensitivity reactions for one hour after completion of the procedure.

Shocks were delivered at low energy levels at the beginning of lithotripsy which was gradually increased to the recommended energy level. The number of shocks varied from 1800 to 4000 during a treatment session. Patients were discharged after completion of the procedure which was the basis of the fulfillment of the discharge criteria and were asked to follow up after one week.

Pain score was measured before the start of the procedure and then assessed immediately before giving intravenous nalbuphine in those who needed augmentation of analgesia during the procedure and post procedure in those who completed the procedure without any need for intravenous nalbuphine. The number of shock waves and shock wave pressures at which nalbuphine was given was also noted.

Patients were compared for mean age, gender, body mass index (BMI), analgesia requirement, the number of shock waves tolerated without analgesia, the total number of shock waves, peak shock wave pressure, and mean pain score. Data was collected on a specified pro forma, entered and analysed using IBM SPSS version 16.0 (New York, USA). Chi-square test was used to analyse statistical significance of categorical variables. The one-way analysis of variance (ANOVA) (least significant difference or LSD) was used to compare different groups with each other. A p-value of <0.05 was considered statistically significant.

## Results

A total of 135 patients with mean age of 38.7 ± 1.31 years were included, 105 (77.8%) were male and 29 (21.5%) were females. There were 45 patients in each group. Demographic features of the three groups were comparable as shown in Table 1.

**Table 1:** Comparison of patient demographics and stone details BMI: Body mass index; DJ: Double J.

	Group A	Group B	Group C
Mean Age	40.87 ± 12.32	36.04 ± 13.20	39.33 ± 13.84
Gender Male Female	11 (24.4%) 34 (75.6%)	5 (11.1%) 40 (88.9%)	14 (31.1%) 31 (68.9%)
Mean BMI	27.07 ± 4.5	25.5 ± 3.40	28.02 ± 6.48
Laterality Left Right	25 (55.6%) 20 (44.4%)	29 (64.4%) 16 (35.6%)	31 (68.9%) 14 (31.1%)
Mean Stone Size	13.06 ± 3.72	12.43 ± 3.59	13.43 ± 3.01
Previous Lithotripsy Yes No	19 (42.2%) 26 (57.8%)	17 (37.8%) 28 (62.2%)	13 (28.9%) 31 (68.9%)
DJ stent Yes No	22 (48.9%) 23 (51.1%)	23 (51.1%) 22 (48.9%)	17 (37.8%) 28 (62.2%)
Opacity Lucent Faintly opaque Opaque	4 (8.9%) 2 (4.4%) 39 (86.7%)	4 (8.9%) 8 (17.8%) 33 (73.3%)	6 (13.3%) 7 (15.6%) 32 (71.1%)
Stone Location Upper pole Middle pole Lower pole Pelvis	6 (13.31%) 7 (15.6%) 21 (46.7%) 11 (24.4%)	5 (11.1%) 13 (28.9%) 19 (42.2%) 8 (17.8%)	4 (8.9%) 7 (15.6%) 21 (46.7%) 13 (28.9%)

Twenty-four (53.3%) patients in group A required intravenous nalbuphine during the procedure compared to 13 (28.9%) patients in group B and 14 (31.1%) patients in group C. Higher energy levels were noted for groups B and C at which intravenous analgesia was required as compared to group A (p-value = 0.000). Similarly, a greater number of shock waves was observed at which intravenous analgesia was required in groups B and C as compared to group A (p-value = 0.085) (Table 2).

**Table 2:** Outcomes of different variables analysed IV: Intravenous.

	Group A	Group B	Group C	p-value
Mean pre-procedure pain score	0.11 ± 0.44	0.13 ± 0.40	0.11 ± 0.38	0.957
Mean number of shock waves at which IV analgesia required	1273.92 ± 570.9	1675.85 ± 454.3	1412.07 ± 448.5	0.085
Mean energy level at which analgesia required (KV)	15.42 ± 0.717	16.62 ± 0.51	16.43 ± 0.85	0.000
Number of patients requiring IV analgesia	24/45 (53.3%)	13/45 (28.9%)	14/45 (31.1%)	0.030
Mean maximum pain score	4.73 ± 1.80	3.69 ± 1.26	3.31 ± 1.93	0.000
Mean total number of shock waves	3617.55 ± 357.63	3606.67 ± 289.51	3555.56 ± 344.80	0.639
Mean maximum energy level (KV)	17.7 ± 0.86	17.7 ± 0.65	17.7 ± 0.63	0.958

There was a significantly lower number of patients requiring intravenous analgesics in groups B and C as compared to group A (p-value A vs B = 0.017 and A vs C = 0.029), while there was no significant difference between groups B vs C (p-value = 0.826) (ANOVA) (Table 3). The mean pain scores in groups A, B, and C were 4.73 ± 1.8, 3.69 ± 1.3, and 3.3 ± 1.9, respectively (Table 2). There was significantly lower pain score in groups B and C as compared to group A (p-value < 0.05) while no significant difference was seen between groups B and C (Table 3).

**Table 3:** Variables analysis among different groups (ANOVA, LSD) ANOVA: Analysis of variance; IV: Intravenous; LSD: Least significant difference.

Variables	Comparison	p-value
Mean number of shock waves at which IV analgesia was required	A vs B A vs C B vs C	0.027 0.426 0.187
Mean energy level at which IV analgesia was required	A vs B A vs C B vs C	0.000 0.000 0.499
Number of patients requiring IV analgesia	A vs B A vs C B vs C	0.017 0.029 0.826
Mean maximum pain score	A vs B A vs C B vs C	0.004 0.000 0.290

## Discussion

After its introduction by Chaussy in 1980, ESWL has become the standard treatment of choice for renal and ureteric stones. It is simple, noninvasive, ambulatory, effective, and associated with minimal comorbidities [1-2]. The pathogenesis of pain during ESWL is not clearly defined yet. It is proposed to be associated with stimulation of nociceptors in skin, renal capsule, pleural parietal peritoneum, and muscles [1, 4].

Factors affecting the analgesia requirement during ESWL are age, gender, the amount of energy used, site and size of the stone, shock wave peak pressure, type of lithotripter, size of the focal zone, and cavitation effects [1, 7]. With the introduction of new models of lithotripters, the trend for anesthesia has progressed from general and regional anesthesia to sedative analgesic techniques [8]. Pain during procedure results in poor compliance. Poor analgesia can make stone targeting more difficult resulting in poor stone-free rates. Thus, a pain-free patient is a better candidate for ESWL to enhance the effectiveness of procedure [7].

Different analgesics including opioids, NSAIDs, local analgesia and different combinations are used via different routes. However, there is as yet no consensus on standard analgesia for pain during ESWL [1, 4-5]. Opioids are the most commonly used analgesics for pain control but they are associated with respiratory depression, bradycardia, hypotension, nausea, vomiting, and an extended monitoring time [9]. Initially, NSAIDs and topical anesthetic creams were used as adjuncts with opioids to reduce their dose and side effects. With time, however, in certain centers, they replaced opioids and started being used as sole agents for pain relief during the procedure [10].

Locally applied analgesic creams have a controversial role for pain control. Advantages include their simplicity, non-invasiveness, avoiding the side effects of intramuscular and intravenous analgesics, and their potential use as coupling medium [11-12]. Their disadvantage is that they are relatively weak analgesics requiring other additional analgesics like opioids and NSAIDs for pain control [13]. In most of the previous studies, eutectic mixture of local analgesia (EMLA) cream (2.5% prilocaine and 2.5% lidocaine) was used as a local analgesic. Considering the fact that it is not easily available in Pakistan and its high cost, we used 2% lidocaine gel, 30 minutes before the procedure, for local analgesia.

Saita, et al. described the role of intramuscular analgesia with locally applied Luan gel (lidocaine gel 1%) to decrease the discomfort during ESWL and to increase the success rate of ESWL for ureteral and renal stones [14]. Vilar, et al., in their analysis, used intravenous pethidine and EMLA cream in one group and intravenous pethidine and placebo cream in another group for pain control during ESWL. They concluded that EMLA cream combined with intravenous pethidine improved the visual analogue score, stone fragmentation, and decreased rate of withdrawal from ESWL procedure versus intravenous pethidine and placebo cream [15]. Similarly, Kumar, et al. showed improved pain control and better stone-free rate with topical EMLA cream in combination with diclofenac sodium as compared to either of the entities used alone [16].

On the other hand, Acar, et al. in their study of 60 patients compared EMLA with placebo cream before ESWL and patient controlled analgesia with remifentanil during the procedure and described no difference in the dose of remifentanil and pain score between the two groups [6]. Eryildirim, et al. too in their analysis of 120 patients compared the effects of EMLA cream alone, intramuscular diclofenac sodium alone, and their combination. They demonstrated that no difference in pain control of patients was noted in patients using diclofenac sodium with or without EMLA cream and instead superior effect of intramuscular diclofenac sodium over EMLA cream was noted [17].

The results of our study are consistent with those of Acar, et al. and Eryildirim, et al. We found no significant difference among mean pain score and the need for supplemental intravenous analgesia between groups taking naproxen sodium with and without lidocaine gel. There was also no statistical difference in energy levels and the number of shockwaves at which intravenous analgesia was required between the aforementioned groups.

NSAIDs are considered more potent analgesics with fewer side effects as compared to opioids for renal colic [18]. They are equally effective as opioids for pain relief during ESWL. They provide effective pain relief through the inhibition of cyclooxygenase 1 (COX-1) and cyclooxygenase 2 (COX-2) enzymes. They are associated with mild gastrointestinal disturbances, occasional hypersensitivity reactions, and occasional coagulation disorders [19]. There are different NSAIDs in use nowadays.

We used naproxen sodium 550 mg per oral 45 minutes before ESWL in our study. Naproxen sodium is one of the most commonly used NSAIDs. It has the lowest risk of vascular events when compared to other NSAIDs [20]. No previous study compared the analgesic effect of naproxen sodium for pain relief in ESWL.

Ozkan, et al. compared the effect of lornoxicam (short acting NSAIDs), paracetamol, and tramadol for pre-procedural analgesia. They concluded that lornoxicam is a better analgesic than either paracetamol or tramadol [21]. Iqbal, et al. in their study described the superior role of intramuscular diclofenac sodium in combination with diclofenac gel as compared to diclofenac gel alone [8]. Issa, et al., in their trial, reported better pain control and lower supplemental analgesia requirement with ketorolac as compared to morphine and EMLA cream [22].

In our study when we used oral naproxen sodium with or without lidocaine gel, there was a significant reduction in both the mean pain score and the need for supplemental intravenous analgesia as compared to the use of locally applied lidocaine gel alone. There were significantly high energy levels at which intravenous analgesia was required in groups B and C as compared to group A. However, approximately one-third of patients in both oral and mixed group needed rescue intravenous analgesia during the procedure.

No patient in our study had any adverse drug reaction thus suggesting that the use of oral naproxen sodium is safe for pain control during ESWL and may be augmented with on demand intravenous analgesia.

## Conclusions

Due to the recent introduction of ESWL and lack of high-quality data regarding pain management strategies during the procedure, no standardised guidelines have been defined for analgesia use for ESWL.

Our study furthered the efforts made by previous investigators in comparing the effect of various analgesia options. A total of three strategies, lidocaine gel alone, oral naproxen alone and a combination of the two, were investigated. The variables being analysed included analgesia requirement, the number of shock waves tolerated without analgesia, the total number of shock waves, peak shock wave pressure, and mean pain score.

Our results demonstrated that there was no significant effect of locally applied 2% lignocaine gel on mean pain score and intravenous analgesia requirement during ESWL procedure. However, there was a statistically significant lower pain score and lower supplemental analgesia requirement in patients taking oral naproxen sodium with or without lidocaine gel when compared with locally applied lignocaine gel alone.

We, therefore, concluded that oral naproxen sodium with on demand intravenous analgesia is a safe and effective option for ESWL related pain. Further studies need to be conducted to compare its use with that of other pain management strategies such as the use of opioids with regards to their effects on the success rates of ESWL, mean pain score, adverse effects as well as the willingness of the patients to undergo the procedure in case of recurrence using the same pain management strategy.

## References

[1] Gupta NP, Kumar A (2008). Analgesia for pain control during extracorporeal shock wave lithotripsy: current status. Indian J Urol.

[2] Al-Marhoon MS, Shareef O, Al-Habsi IS (2013). Extracorporeal shock-wave lithotripsy success rate and complications: initial experience at Sultan Qaboos University Hospital. Oman Med J.

[3] Mazdak H, Abazari P, Ghassami F (2007). The analgesic effect of inhalational Entonox for extracorporeal shock wave lithotripsy. Urol Res.

[4] Bach C, Zaman F, Kachrilas S (2011). Drugs for pain management in shock wave lithotripsy. Pain Res Treat.

[5] Yesil S, Polat F, Ozturk U (2014). Effect of different analgesics on pain relief during extracorporeal shock wave lithotripsy. Hippokratia.

[6] Acar A, Erhan E, Nuri Deniz M (2013). The effect of EMLA cream on patient-controlled analgesia with remifentanil in ESWL procedure: a placebo-controlled randomized study. Anesth Pain Med.

[7] Young A, Ismail M, Papatsoris A (2012). Entonox® inhalation to reduce pain in common diagnostic and therapeutic outpatient urological procedures: a review of the evidence. Ann R Coll Surg Engl.

[8] Iqbal J, Pall M, Mette UK (2013). Analgesia for ESWL: comparing two analgesia techniques. A double blind randomized study. Internet J Urol.

[9] Demir A, Cecen K, Karadag MA (2015). Pain control using pethidine in combination with diazepam compared to diclofenac in combination with hyoscine-n-butyl bromide: in patients undergoing extracorporeal shock wave lithotripsy. Cent European J Urol.

[10] Cohen E, Hafner R, Rotenberg Z (1998). Comparison of ketorolac and diclofenac in the treatment of renal colic. Eur J Clin Pharmacol.

[11] Dawson C, Vale JA, Corry DA (1994). Choosing the correct pain relief for extracorporeal lithotripsy. Br J Urol.

[12] Honnens de Lichtenberg M, Miskowiak J, Mogensen P (1992). Local anesthesia for extracorporeal shock wave lithotripsy: a study comparing eutetic mixture of local anesthetics cream and lidocaine infiltration. J Urol.

[13] Basar H, Yilmaz E, Ozcan S (2003). Four analgesic techniques for shockwave lithotripsy: eutectic mixture local anesthetic is a good alternative. J Endourol.

[14] Saita A, Bonaccorsi A, Aquilino M (2004). ESWL: comparing two analgesic techniques. Our experience. Urol Int.

[15] Vilar DG, Fadrique GG, Sacoto CD (2012). Topical EMLA for pain control during extracorporeal shock wave lithotripsy: prospective, comparative, randomized, double-blind study. Urol Res.

[16] Kumar A,  Gupta NP,  Hemal AK (2007). Comparison of three analgesic regimens for pain control during shockwave lithotripsy using Dornier Delta Compact lithotripter: a randomized clinical trial. J Endourol.

[17] Eryildirim B, Kuyumcuoglu U, Tarhan F (2009). Comparison of three analgesic treatment protocols for pain management during extracorporeal shock wave lithotripsy. Urol Int.

[18] Holdgate A, Pollock T (2004). Systematic review of the relative efficacy of non-steroidal anti-inflammatory drugs and opioids in the treatment of acute renal colic. BMJ.

[19] Mezentsev VA (2009). Meta-analysis of the efficacy of non-steroidal anti-inflammatory drugs vs. opioids for SWL using modern electromagnetic lithotripters. Int. braz j urol.

[20] Kearney PM, Baigent C, Godwin J (2006). Do selective cyclo-oxygenase-2 inhibitors and traditional non-steroidal anti-inflammatory drugs increase the risk of atherothrombosis? Meta-analysis of randomised trials. BMJ.

[21] Ozkan F,  Erdemir F,  Erkorkmaz U (2012). Comparison of three different analgesic protocols during shockwave lithotripsy. J Endourol.

[22] Issa MM,  El-Galley R,  McNamara DE (1999). Analgesia during extracorporeal shock wave lithotripsy using the Medstone STS lithotriptor: a randomized prospective study. Urology.

